# Killian-Jamieson diverticulum safely resected using a manual intraoperative neural monitoring system: a case report

**DOI:** 10.1186/s40792-020-00805-0

**Published:** 2020-02-26

**Authors:** Ryo Ataka, Shigeru Tsunoda, Saori Goto, Tatsuto Nishigori, Shigeo Hisamori, Kazutaka Obama, Yoshiharu Sakai

**Affiliations:** Department of Surgery, Graduate School of Medicine, Kyoto University, 54 Kawahara-cho, Shogoin, Sakyo-ku, Kyoto, 606-8507 Japan

**Keywords:** Killian-Jamieson diverticulum, Pharyngoesophageal diverticulum, Intraoperative nerve monitoring, Recurrent laryngeal nerve

## Abstract

**Background:**

Killian-Jamieson diverticulum is a rare pharyngoesophageal diverticulum. The risk of intraoperative injury of the recurrent laryngeal nerve (RLN) is high during surgical resection of Killian-Jamieson diverticulum because the RLN usually runs next to the base of the diverticulum. We present a case of Killian-Jamieson diverticulum that was safely resected with effective use of an intraoperative nerve monitoring (IONM) system with a handheld stimulating probe to prevent RLN injury.

**Case presentation:**

A 69-year-old man complaining of dysphagia was diagnosed with Killian-Jamieson diverticulum and underwent open transcervical diverticulectomy. Because the anterior aspect of the diverticulum was expected to be close to the RLN, the accurate location of the RLN was checked during dissection by intermittent stimulation using a handheld probe of the IONM system to avoid mechanical and thermal injury. The diverticulum was transected longitudinally using a linear stapler, and the staple line was buried using absorbable sutures from the distal end. During its closure, RLN was identified very close to the diverticulum stump by IONM, and the upper side of the stump was left unburied to avoid RLN injury. The postoperative course was uneventful and the patient was discharged on postoperative day 7. Postoperative evaluation showed no vocal cord paralysis.

**Conclusion:**

IONM may be beneficial during open surgery for Killian-Jamieson diverticulum, which usually protrudes just lateral to the RLN.

## Background

Pharyngoesophageal diverticulum is one of the rarest intestinal diverticula. Killian-Jamieson diverticulum is much more uncommon than Zenker’s diverticulum [1]. This pathology is characterized by the unique anatomical abnormality, laterally arising from the Killian-Jamieson area, a space just below the cricopharyngeus muscle, while Zenker’s diverticulum arises from the posterior wall of cervical esophagus just above the cricopharyngeus muscle. Due to the anatomical location, the risk of intraoperative injury to the recurrent laryngeal nerve (RLN) is higher with Killian-Jamieson diverticulum than with Zenker’s diverticulum. We present a rare case of Killian-Jamieson diverticulum that was safely resected with the effective use of an intraoperative nerve monitoring (IONM) system with a handheld stimulating probe to prevent RLN injury.

### Case presentation

**Fig. 1:**
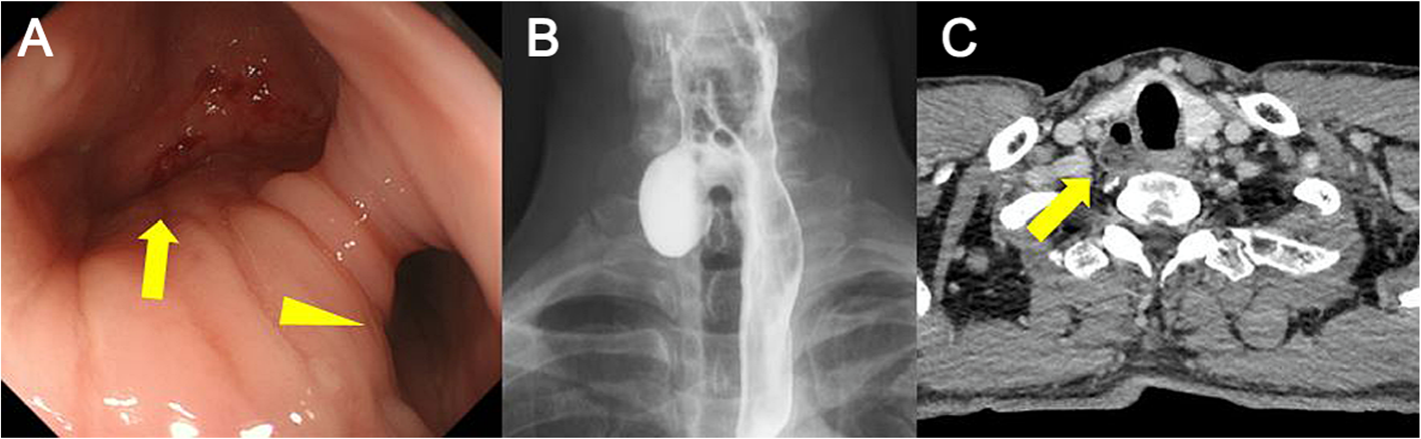
**a** Upper intestinal endoscopy shows a diverticulum (yellow arrow) and the true lumen of the esophagus (yellow arrowhead). **b** Esophagogram shows a right-sided outpouching sac from the esophagus. **c** Computed tomography with contrast shows diverticulum from the esophagus at the level of the cricothyroid cartilage

A 69-year-old Japanese man was referred to our hospital with a chief complaint of discomfort during swallowing over the past few years. He was on anticoagulant, antiarrhythmic, and antihyperlipidemic medication for paroxysmal atrial fibrillation and dyslipidemia. Physical examination revealed no abnormality, and results of laboratory tests were unremarkable. Upper endoscopy showed diverticulum at the cervical esophagus filled with food debris. The mucosa showed slightly reddish discoloration due to chronic inflammation (Fig. 1a), visualized as a brownish area under narrow-band imaging. Biopsy provided no evidence of malignancy. Barium esophagogram also showed a right-sided outpouching sac from the esophagus (Fig. 1b). Contrast-enhanced computed tomography showed a 30-mm diverticulum behind the right thyroid lobe, protruding laterally from the esophagus at the level of the cricothyroid cartilage (Fig. 1c). Based on these findings, Killian-Jamieson diverticulum was diagnosed.

**Fig. 2:**
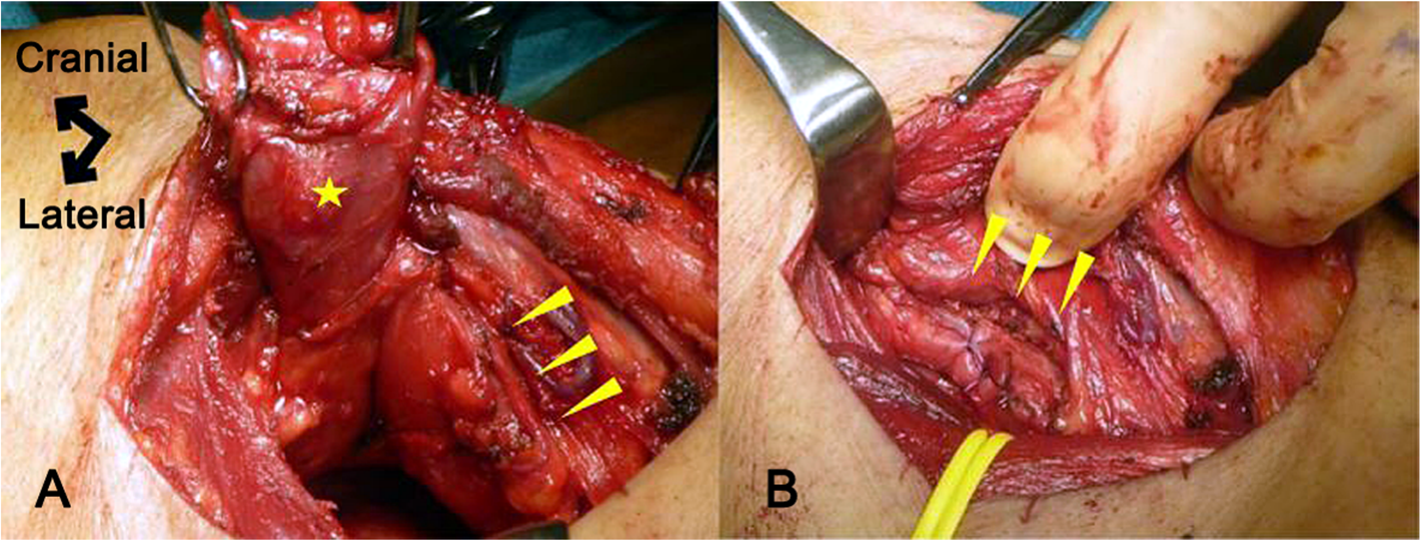
Intraoperative pictures before and after resection of the diverticulum. **a** Diverticulum with a defective area of muscular layer (star) and the area of the right recurrent laryngeal nerve (arrowheads). **b** Area of the right inferior laryngeal nerve (arrowheads) and nearby sutured longitudinal muscles

**Fig. 3:**
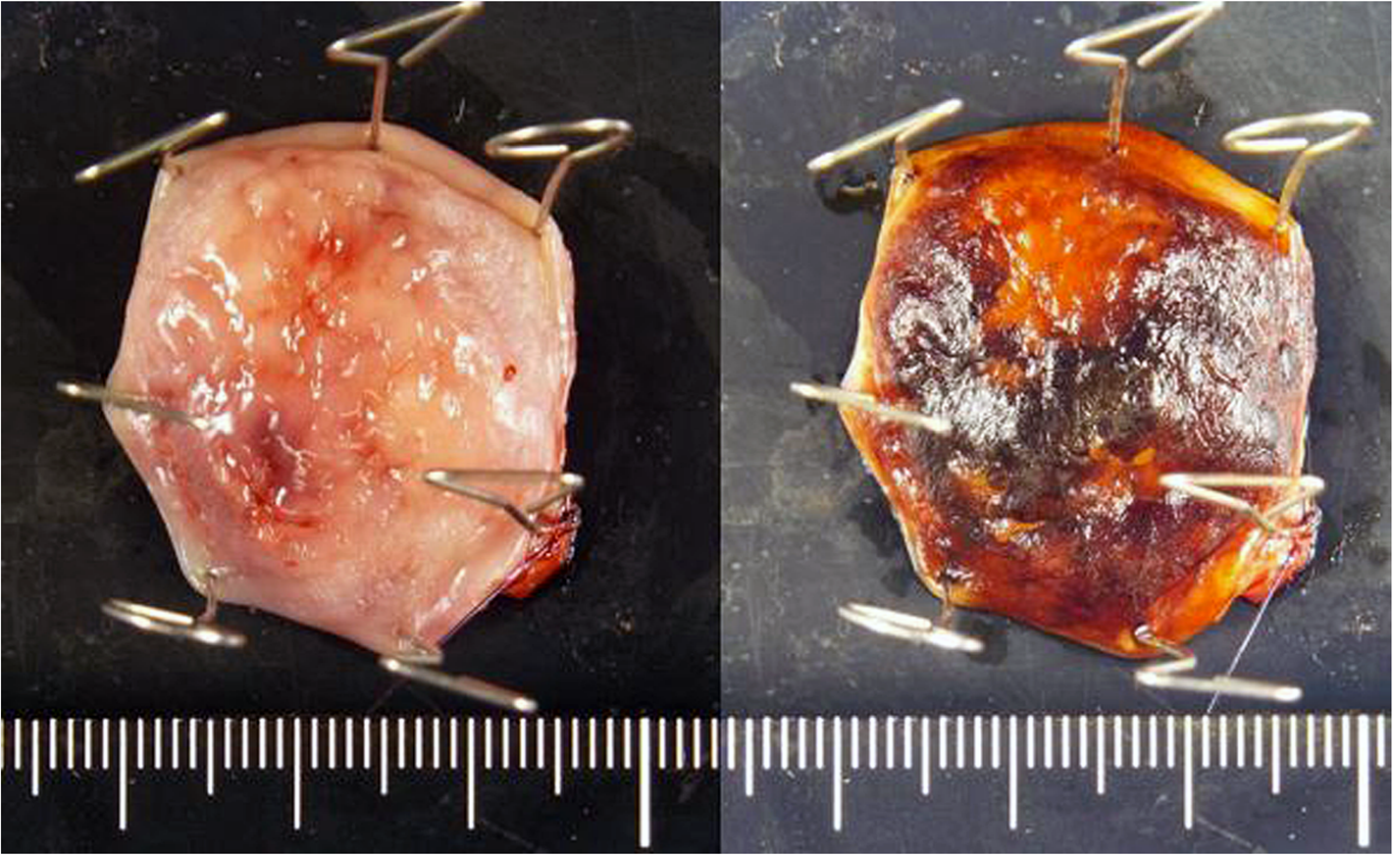
Specimen pictures with or without iodine staining

Transcervical diverticulectomy was performed under general anesthesia. The cervical esophagus was accessed through a right oblique incision, and the white thickened diverticulum without a covering muscle layer was identified without difficulty. In the meantime, the right vagus nerve was identified and taped for nerve monitoring. An IONM system (Nerve Integrity Monitoring Response 3.0 system; Medtronic, Japan) with a handheld stimulating probe was used to confirm the intact electrophysiologic connection between the right vagus nerve and right vocal cord. The diverticulum was carefully dissected from the surrounding tissue. Because the anterior aspect of the diverticulum was expected close to the RLN, the accurate location of the RLN was checked during dissection by intermittent stimulation using the handheld probe to avoid mechanical or thermal injury. Finally, the base of the diverticulum was exposed below the cricopharyngeus muscle and just lateral to longitudinal esophageal muscle, confirming the diagnosis of Killian-Jamieson diverticulum. The diverticulum was then transected longitudinally using a linear stapler, and the staple line was buried using absorbable sutures from the distal end. During its closure, the RLN coursed very close to the diverticulum stump according to IONM and burying the entire stump proved impossible (Fig. 2). Histopathological diagnosis of the specimen was pseudodiverticulum without a muscular layer, compatible with Killian-Jamieson diverticulum. No evidence of malignancy was found despite chronic inflammatory changes (Fig. 3).

**Fig. 4:**
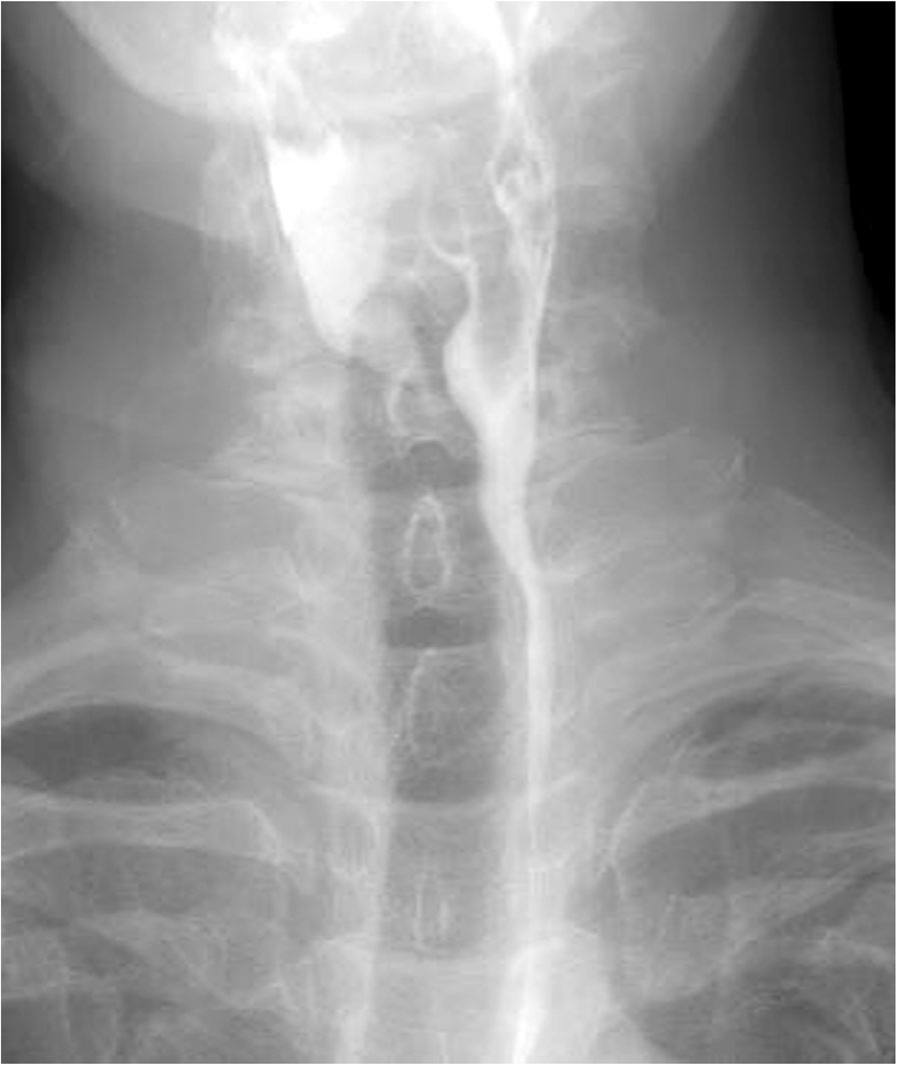
Postoperative esophagogram reveals no remnant diverticulum

The postoperative course was uneventful. Postoperative esophagography confirmed complete resection of the diverticulum (Fig. 4). Laryngoscopy by the otorhinolaryngologist confirmed intact vocal cord mobility on postoperative day 4. Oral feeding was resumed on postoperative day 4, and the patient was discharged on postoperative day 7. As of the time of writing, no recurrence or discomfort during swallowing has been seen postoperatively.

## Discussion

Pharyngoesophageal diverticulum was first described by Ludilow in 1767 [2], and is now classified into three types: Zenker’s diverticulum, Killian-Jamieson diverticulum, and Laimer’s diverticulum. Pharyngoesophageal diverticulum is one of the rarest intestinal diverticula, and the incidence of Zenker’s diverticulum, as the most common type, has been reported as 2 per 100,000 capita per year [3].

**Fig. 5:**
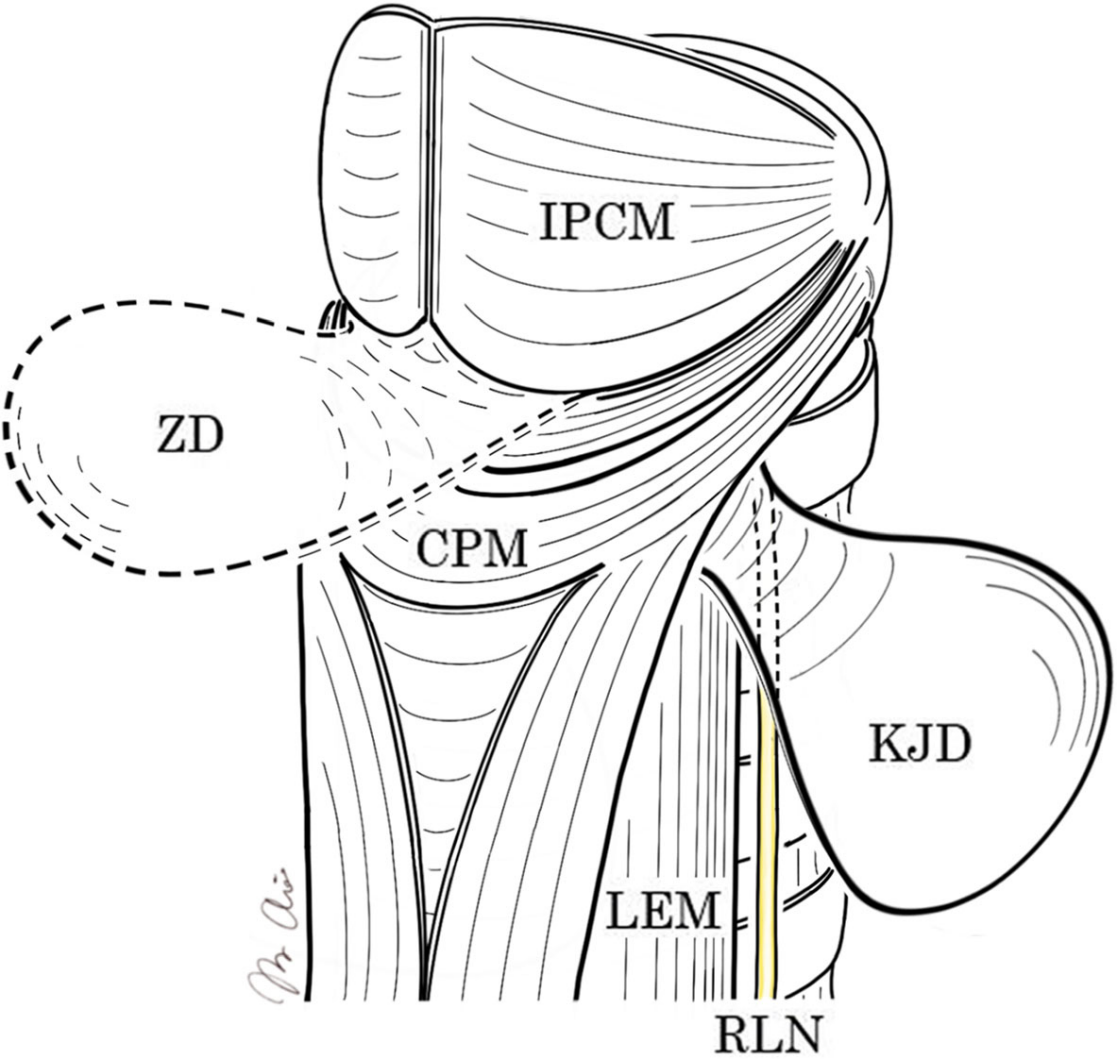
Anatomical structure of the hypopharynx and cervical esophagus. ZD, Zenker’s diverticulum; KJD, Killian-Jamieson diverticulum; RLN, recurrent laryngeal nerve; IPCM, inferior pharyngeal constrictor muscle; CPM, cricopharyngeus muscle; LEM, longitudinal esophageal muscle

Three anatomically weak areas are known where the pulsion diverticula potentially arise. Killian-Jamieson diverticulum was first reported by Ekberg and Nylander in 1983 [4], and the incidence has been reported as one quarter of that of Zenker’s diverticulum [1]. The diverticulum arises laterally from the Killian-Jamieson area, a space just below the cricopharyngeus muscle and lateral to the esophageal longitudinal muscle (Fig. 5).

Management of the pharyngoesophageal diverticulum depends on clinical symptoms including pharyngitis, dysphasia, hoarseness, and vomiting. In general, surgery is indicated only in symptomatic cases. However, it should be kept in mind that some pharyngoesophageal diverticula may involve malignant lesions. Zenker’s diverticulum has been reported to develop esophageal malignancy in 0.4~1.5% of cases [5], while no cases of Killian-Jamieson diverticulum with malignancy have been reported. Although open surgical resection was regarded as a preferable option for Zenker’s diverticulum when compared to flexible endoscopic resection in a systematic review [6], the optimal procedure for Killian-Jamieson diverticulum remains unclear because of the limited reports of cases of this rare condition. The risk of RLN injury is supposed to be higher for Killian-Jamieson diverticulum than for Zenker’s diverticulum because of the anatomical relationships (Fig. 5). Open transcervical excision rather than endoscopic resection thus appears to offer safer management. Some authors have concluded that endoscopic stapling can provide a safe and effective choice of treatment for Killian-Jamieson diverticulum [7, 8], but the technique should be performed only in experienced centers or in selected cases in which general anesthesia cannot be tolerated.

IONM has been developed over the past 30 years and has been clinically applied for about 20 years. Several authors have recently reported IONM as a safe, reliable method to prevent nerve injury during thyroid, parathyroid, laryngeal, or esophageal surgery [9–13]. Thanks to that system, operators are able to detect and preserve the RLN or superior laryngeal nerve more easily and safely. RLN monitoring consists of a nerve stimulator and a laryngeal electromyography (EMG) endotracheal tube (NIM TriVantage EMG endotracheal tube; Medtronic). IONM monitors EMG activity from the vocal cords in two ways: continuous automatic monitoring using automatic periodic stimulation (APS) electrodes, and intermittent manual monitoring using handheld stimulating probes. In both methods, once a particular nerve is activated or stimulated, the NIM system monitors EMG activities and changes from muscles innervated by the affected nerve, allowing the surgeon to alter maneuvers to minimize intraoperative trauma to the nerve. Few cases of Killian-Jamieson diverticulum safely resected using an IONM that have been described [11]. In the present case, we applied a manual IONM with a handheld stimulating probe to test any point we want to check without directly exposing the RLN. We were able to confirm the location of the right inferior laryngeal nerve for intact preservation during dissection and closure of the longitudinal muscular layer. Of note, in the latter procedure, the RLN was identified closer than expected to the esophageal wall after thorough dissection of the diverticulum, which might derange the normal anatomical location.

As shown in the present case, IONM systems offer a good method to preserve the RLN during benign esophageal surgery. For the best use of IONM systems, understanding differences between continuous automatic monitoring by APS electrode and intermittent manual IONM is very important. The former is a simple, easy procedure enabling early detection of inadequate neural traction due to rough surgical procedures, while the latter enables more precise localization of nerve tracts. However, two serious complications were reported following the use of continuous automatic monitoring. The one was temporary true vocal fold hypomobility due to traumatic dislodgement of the vagal electrode, and the other was hemodynamic instability caused by autonomic neural imbalance such as bradycardia and hypotension [14]. Therefore, the intermittent manual IONM is considered more appropriate in anatomically complicated cases such as Killian-Jamieson diverticulum or large Zenker’s diverticulum. Regarding the demerit of the use of IONM, muscle relaxation must be minimized or eliminated to optimize IONM [15], and the equipment requires extra cost, which may be justified considering the rarity of Killian-Jamieson diverticulum.

## Conclusion

We have presented a rare case of Killian-Jamieson diverticulum that was safely resected by open transcervical diverticulectomy with manual IONM. The IONM may be beneficial during open operations for Killian-Jamieson diverticulum, which usually protrudes just lateral to the RLN.

## Data Availability

Data sharing is not applicable to this article as no datasets were generated or analyzed during the current study.

## Abbreviations

RLN: Recurrent laryngeal nerve
IONM: Intraoperative nerve monitoring
EMG: Electromyography
APS: Automatic periodic stimulation
